# Effect of Correlated tRNA Abundances on Translation Errors and Evolution of Codon Usage Bias

**DOI:** 10.1371/journal.pgen.1001128

**Published:** 2010-09-16

**Authors:** Premal Shah, Michael A. Gilchrist

**Affiliations:** 1 Department of Ecology & Evolutionary Biology, University of Tennessee, Knoxville, Tennessee, United States of America; 2 National Institute for Mathematical and Biological Synthesis, University of Tennessee, Knoxville, Tennessee, United States of America; Harvard University, United States of America

## Abstract

Despite the fact that tRNA abundances are thought to play a major role in determining translation error rates, their distribution across the genetic code and the resulting implications have received little attention. In general, studies of codon usage bias (CUB) assume that codons with higher tRNA abundance have lower missense error rates. Using a model of protein translation based on tRNA competition and intra-ribosomal kinetics, we show that this assumption can be violated when tRNA abundances are positively correlated across the genetic code. Examining the distribution of tRNA abundances across 73 bacterial genomes from 20 different genera, we find a consistent positive correlation between tRNA abundances across the genetic code. This work challenges one of the fundamental assumptions made in over 30 years of research on CUB that codons with higher tRNA abundances have lower missense error rates and that missense errors are the primary selective force responsible for CUB.

## Author Summary

Codon usage bias (CUB) is a ubiquitous and important phenomenon. CUB is thought to be driven primarily due to selection against missense errors. For over 30 years, the standard model of translation errors has implicitly assumed that the relationship between translation errors and tRNA abundances are inversely related. This is based on an implicit and unstated assumption that the distribution of tRNA abundances across the genetic code are uncorrelated. Examining these abundance distributions across 73 bacterial genomes from 20 different genera, we find a consistent positive correlation between tRNA abundances across the genetic code. We further show that codons with higher tRNA abundances are not always “optimal” with respect to reducing the missense error rate and hence cannot explain the observed patterns of CUB.

## Introduction

Protein production is the most energetically expensive metabolic process within a cell [1]–[4]. However, like all biological processes, protein translation is prone to errors. The biological importance of these translation errors and their impact on coding sequence evolution, especially the evolution of codon usage bias (CUB), depends on both their effects on protein function and their frequencies. Translation errors fall into two categories: nonsense errors and missense errors. Nonsense errors, also referred to as processivity errors, occur when a ribosome prematurely terminates translating a coding sequence. Missense errors occur when the wrong amino acid is incorporated into a growing peptide chain. Although many possible factors such as mRNA stability and recombination likely contribute to the evolution of CUB, selection against translation errors and biased mutation are thought to be the primary forces [5]–[11].

Most researchers believe that CUB results primarily from selection against missense errors or, equivalently, for translational accuracy (see [10], [12]–[15]). In addition to limited empirical observations, the main evidence cited as supporting this belief includes the fact that preferred synonymous codons (i.e. the codons over-represented in high expression genes) have higher cognate tRNA abundances and that these codons are also favored at evolutionarily conserved sites [12], [13]. While the preferred codons may indeed be ‘optimal’ in some limited sense, as we demonstrate below, the idea that they minimize missense error rates is based on an overly simplistic understanding of the relationship between tRNA abundances and missense error rates.

The effect of missense errors on protein function is equivalent to a non-synonymous point mutation. Because amino acids with similar properties are clustered within the genetic code [16]–[19], the genetic code is generally considered to be adapted to minimize the *phenotypic effects* of point mutations and missense errors. However, despite its importance, the adaptedness of tRNA abundances across the genetic code to reduce the *rate* of translation errors has received almost no attention. For instance, in *E. coli* the average nonsense and missense error rates are estimated to be on the order of 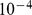 to 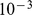 per codon, respectively [10], [20]–[25]. This implies that for an average length gene of 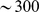 amino acids, about 3–26% of its protein products will contain at least one translation error. However, since the only available estimates of missense error rates are for specific amino acid misincorporations [20]–[22], these rates are likely gross underestimates as they do not take into account all possible amino acid misincorporations at that codon.

Currently, missense errors are thought to be the result of competition between tRNAs with the right amino acid (cognates) and the ones with the wrong amino acids (near-cognates) for the codon at the ribosomal *A*-site [25]–[27]. A near-cognate tRNA is characterized by a single codon-anticodon nucleotide mismatch and codes for an amino acid different from that of the *A*-site codon [28]–[30]. As a result of this competition, the rate of missense errors at a codon should be strongly affected by the abundances of both cognate and near-cognate tRNAs [25]. For example, an increase in cognate tRNA abundances is predicted to lead to a decrease in a codon's missense error rate. In contrast, an increase in near-cognate tRNA abundances is predicted to lead to an increase in a codon's missense error rate [25].

Previous studies of CUB have generally assumed that amongst a set of synonymous codons, the one with the correspondingly highest tRNA abundance is the one with the lowest missense error rate. However, because missense error rates are thought to be a function of *both* cognate and near-cognate tRNA abundances, if tRNA abundances are positively correlated across the genetic code this assumption may not hold. In this study we ask a fundamental question, “Are tRNA abundances correlated across the genetic code?” Finding that tRNA abundances are indeed generally positively correlated across a wide range of prokaryotes, we then ask, “How does the distribution of tRNA abundances affect the relationship between codon translation and error rates?” This question is of critical importance because the currently favored explanation of CUB, what we will refer to as the standard model, implicitly assumes that codons with the highest translation rates are also the ones with the lowest missense error rates. Our results indicate that this basic assumption only holds for a limited subset of amino acids. As a result, our work strongly suggests that missense errors play a smaller role in the evolution of CUB than currently believed and that the observed patterns of codon conservation observed by Akashi and others are likely due to other selective forces such as selection for translational efficiency or against nonsense errors.

## Results

**Figure 1:**
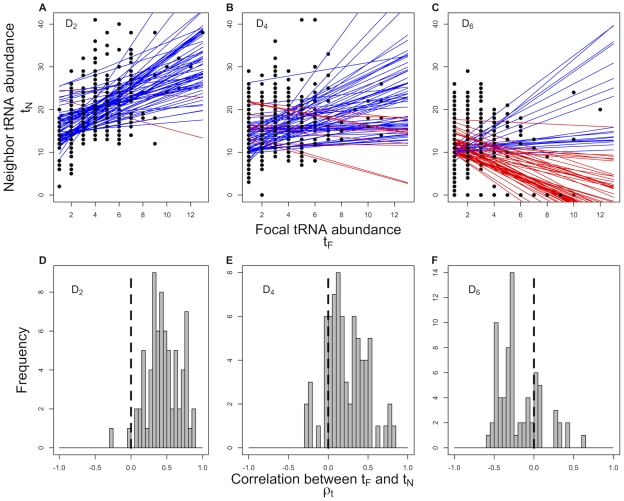
Correlation between a focal tRNA's abundance 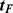 and the abundance of its neighbors 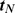, 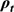 across 73 prokaryotic genomes. Each point in panels (A–C) represents a tRNA species that encodes an amino acid with degeneracy 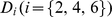. The solid lines represent the regression lines between 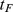 and 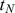 for each genome. Genomes with a negative 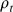 are coded in red, while genomes with a positive 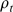 are represented by blue lines. Panels (D–F) present the distribution of correlation coefficients 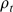 between 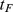 and 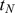 across all the genomes. The mean of the distribution of 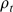 values for all the three degenerate classes differ significantly from 0 (Wilcox test, 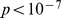).

We began our analysis by first assuming that the abundance of a tRNA species within a cell is proportional to its gene copy number (GCN). This relationship between tRNA abundance and GCN is often made in studies of CUB and has been observed in both prokaryotes and eukaryotes [8], [31], [32]. We obtained GCNs of each tRNA type within an organism from the Genomic tRNA Database GtRNAdb [33] for 73 bacterial genomes representing 50 species from 20 genera (see Table S1 for list of genomes analyzed). We classified each amino acid based on its level of degeneracy 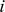, where 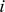 represents the number of synonymous codons of that amino acid. As a result, each amino acid is placed in one of five different degenerate categories 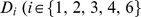). For instance, alanine belongs to the 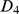 class, while lysine belongs to the 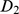 class as these amino acids are coded by 4 and 2 codons, respectively. Serine represents a special case as it is encoded by two disjoint degenerate subsets. As a result we treated each of these subsets as a separate amino acid. We calculated the correlation between GCN of a focal tRNA 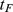 and the sum of GCNs of neighboring tRNAs that coded for a different amino acid and differed from the focal tRNA's anticodon by a single base-pair, 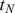 (Table 1). Figure 1 shows the distribution of correlation coefficients 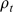 between 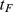 and 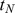 for three degenerate classes of amino acids 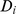 within each of the genomes we examined.

**Table 1:** List of symbols.

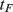	tRNA gene copy number of a focal codon
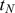	tRNA gene copy number of focal codon's neighbors
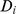	Set of amino acids with 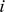 synonymous codons
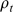	Correlation coefficient between 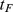 and 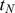
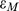	Missense error rate
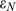	Nonsense error rate
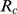	Cognate elongation rate
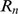	Near-cognate elongation rate
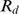	Ribosomal drop-off rate
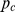	Probability of elongation by cognate tRNA per tRNA entry
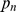	Probability of elongation by near-cognate tRNA per tRNA entry
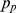	Probability of elongation by pseudo-cognate tRNA per tRNA entry
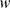	Wobble parameter

We find that the vast majority of genomes (69 out of 73 or 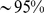) show a positive relationship between the abundance of a focal tRNA species 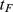 and its one-step non-synonymous neighbors 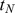, 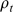 (Binomial test, 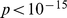, Figure S1). This indicates that tRNAs with similar abundances are closer to each other in the genetic code than expected under the implicit assumptions of the standard model. In other words, according to the standard model the tRNA abundances within the genetic code are predicted to be uncorrelated and the distributions of correlation coefficients 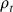 in Figures 1 (d)–(f) are expected to be centered around 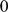. However, we find that under each of the degenerate classes of amino acids, 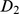, 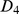 and 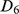, the distribution of 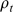 is significantly different from 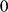 (Wilcox test, 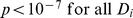). Interestingly, we also find that the distribution of 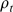 differs considerably between degenerate classes of amino acids. tRNAs corresponding to amino acids in both 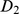 and 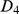 degenerate classes show a significant bias towards a positive correlation between 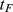 and 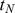, whereas tRNAs in 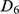 degenerate class are biased towards a negative correlation.

Since the frequency of amino acid usage within a genome is highly correlated with tRNA gene copy number (e.g. in *E. coli* 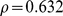, 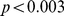), the observed correlations may be the indirect result of amino acid usage bias. In addition to amino acid usage biases, the stereochemistry of codon-anticodon interactions forbids the existence of certain tRNA types [34], potentially contributing to the observed positive correlation among tRNA abundances. In order to address these inherent constraints on the distribution of tRNAs within the genetic code, we randomly distributed tRNA gene copies taking into account the stereochemical constraints, both with and without biased amino acid usage (see Figures S3 and S4). We find that the observed distribution of 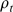 is significantly different from this more complex null distribution for all of the degenerate classes (Kolmogorov-Smirnov test 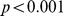 for all cases).

The distribution of tRNAs within the genetic code have important consequences with respect to translation errors and bias in codon usage. Codons with higher tRNA abundances than their coding synonyms are often referred to as ‘optimal’ codons [10] assuming they lead to fewer translation errors [12], [25], [35]. In light of the above results, we now ask the question, “Given that tRNA abundances are positively correlated in the genetic code, do higher cognate tRNA abundances always lead to fewer translation errors?”

### Modeling translation errors

**Figure 2:**
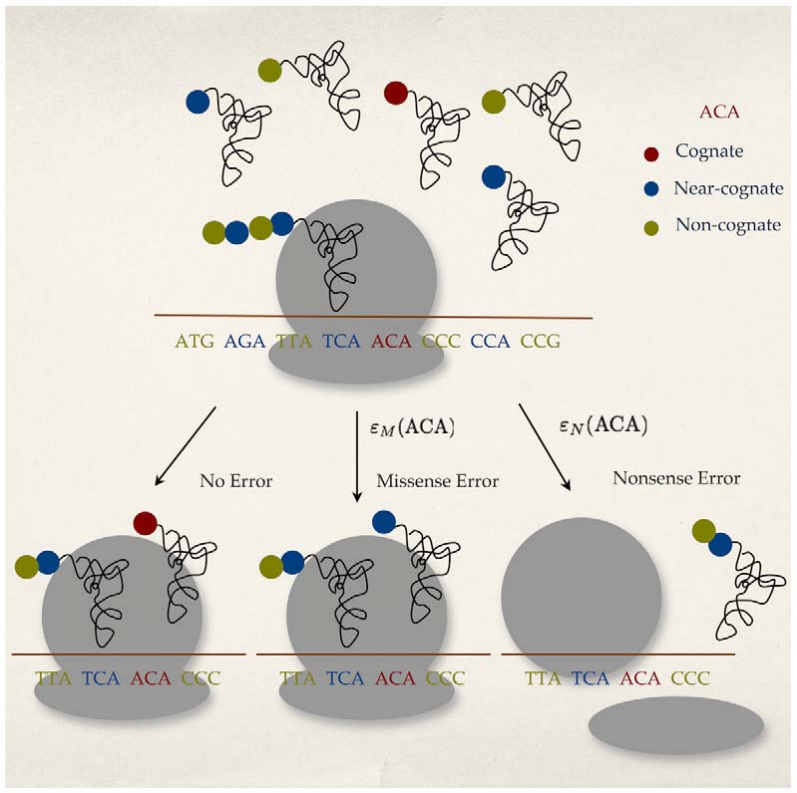
Model of translation errors. During translation, a ribosome pauses at a codon (ACA in this case) waiting for a cognate tRNA. During this pause, one of the three processes can take place: elongation by cognate tRNAs leading to no translation error, elongation by a near-cognate tRNA leading to a missense error with rate 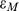 or premature termination of translation due to recognition by release factors, spontaneous ribosome drop-off or frameshifting leading to a nonsense error with a rate 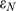.

Following [29], our model of translation errors takes into account competition between cognate and near-cognate tRNAs for the ribosomal *A*-site during translation. We also consider the kinetics of tRNA selection within a ribosome [27] and the effect of codon-anticodon wobble on these kinetics [36]. During protein translation, when a ribosome waits at a given codon, one of three outcomes is likely to occur: (a) elongation by cognate tRNA, (b) elongation by a near-cognate tRNA leading to a missense error or (c) spontaneous ribosomal drop-off, frameshift or recognition by release factors, any of which will lead to a nonsense error (Figure 2). The relative frequency of each of these outcomes determines the rates of missense and nonsense errors at a particular codon.

Assuming an exponential waiting process for a tRNA at codon 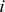, the codon specific missense and nonsense error rates, 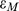 and 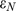 respectively, can be calculated as follows,
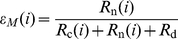
(1)

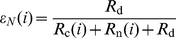
(2)where 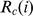 is the codon specific cognate elongation rate, 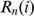 is the codon specific near-cognate elongation rate, and 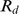 represents the background nonsense error rate (see *Methods* for details).

Using Equations (1) and (2), we calculated codon-specific missense and nonsense error rates for each bacterial genome. In order to understand the effect of codon degeneracy on the relationship between error rates and codon elongation rates, we categorized amino acids based on the number of their synonymous codons 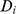 as before. Given our model was parametrized from data on *E. coli*, we also checked for the sensitivity of our analysis to changes in these parameters when extending it to other prokaryotes (Text S1 B).

### Error rates vs. elongation rates

Using *E. coli* strain K12/DH10B (K12) as an example, our estimates of codon-specific missense error rates 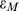 ranged from 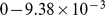 with a median of 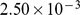. Six of the 61 sense codons have a predicted missense error rate of 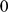 as these codons have no near-cognate tRNA species (Table S2). These rates are higher than recent empirical estimates of missense error rates in *E. coli*, which vary from 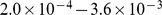 with a median value of 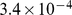 [25]. This is likely due to the fact that the missense error estimates in [25] were for specific amino acid misincorporations, whereas, the values predicted here indicate the rate of all possible missense errors at a given codon. Our predicted rates of codon-specific nonsense errors 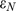 in *E. coli* ranged from 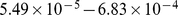 with a median of 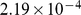 (Table S2).

**Figure 3:**
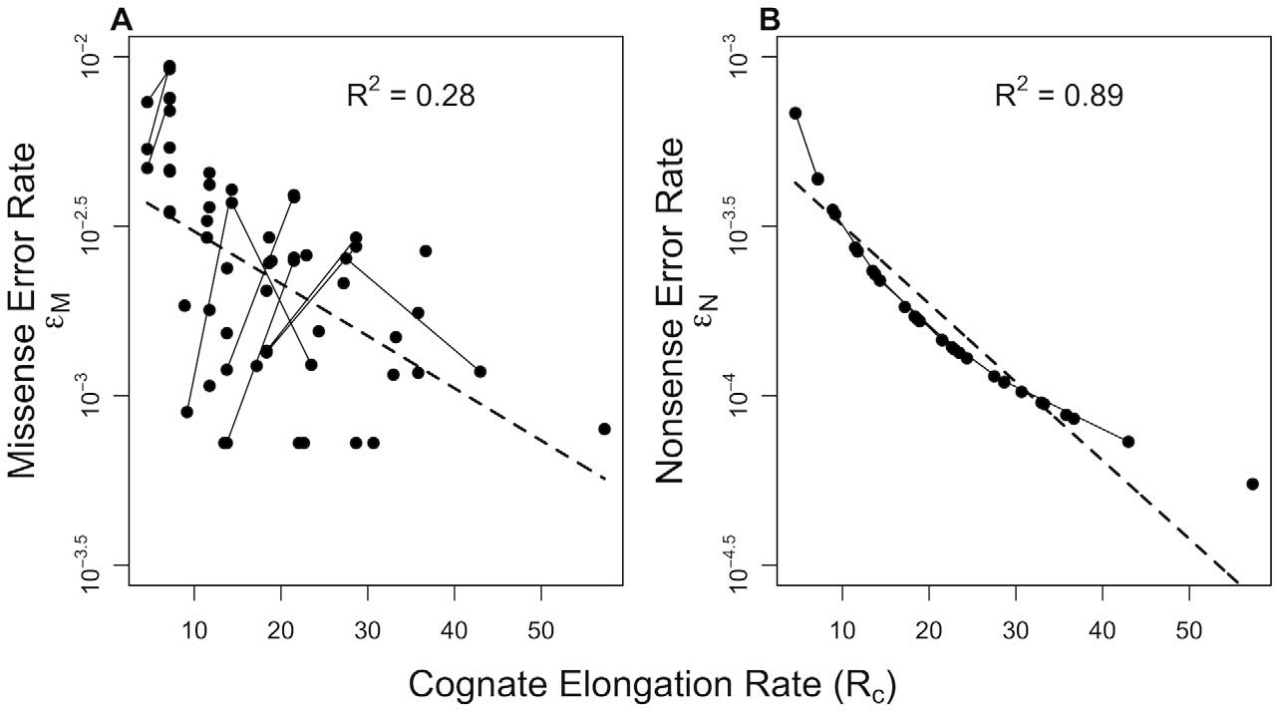
Correlation of translation error rates 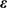 with cognate elongation rate 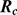 in *E. coli*. We find that rates of both (A) missense 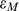 and (B) nonsense errors 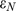 are negatively correlated with the rate of elongation by cognate tRNAs at that codon. The dashed line indicates the regression line between 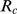 and 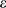. This is consistent with expectations under the standard model. However, in the case of twofold degenerate amino acids (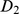), whose two codons are joined together by solid lines, we see that 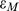 increases with 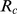 for 8 out of 10 amino acids. In the case of 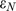 every amino acid showed a decrease in 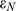 with 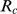.

We find that on average both missense 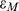 and nonsense error rates 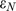 decrease with an increase in cognate elongation rates 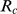 (Figure 3). These results seem, on first glance, largely consistent with the standard model for inferring translation errors from tRNA abundances, which assumes that 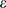 decreases with 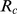. However, because 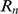 varies between synonymous codons, for about half of the amino acids (10 out of 21) 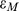 is actually greater for the codon with the highest 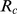 value. This holds even when empirical estimates of tRNA abundances in *E. coli* [31] are used instead of tRNA gene copy numbers (see Figure S5). This result is *inconsistent* with expectations under the standard model that implicitly assumes a codon-independent rate of elongation by near-cognate tRNAs, 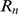. If the abundance of a focal tRNA 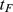 and its neighbors 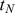 are uncorrelated, then the only factor that affects 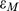 is 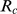. However, as shown earlier, 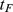 and 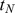 are positively correlated (Figure 1). Thus, the estimates of 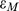 of synonymous codons of an amino acid depend not only on their individual 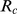 but also on the slope of the relationship between 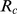 and 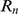. If the rate of increase of 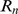 with 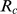 is higher than the relative increase in 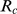, then codons with higher cognate elongation rates 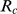 are expected to have *higher* missense error rates 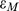 (Figure S2). Interestingly, 8 out of the 10 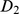 amino acids in *E. coli* K12 showed a positive relationship between 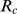 and 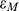. Specifically, we would expect 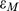 to increase with 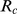 whenever the condition 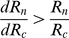 is satisfied. Thus, among the synonymous codons of an amino acid in *E. coli*, the codon with the lowest 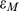 is often not the codon with the highest 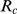. This points to a fundamental change in our understanding of the relationship between tRNA abundances and missense errors and which codons minimize their occurrence.

Interestingly, these results are also consistent with the limited empirical estimates of codon-specific missense error rates. For instance, [22] used *E. coli* to estimate rates at which the asparagine codons AAC and AAU were mistranslated by 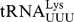. As expected, the authors found that the AAC codon, with a higher 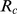 had a lower rate of mistranslation by 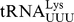 than AAU, with a lower 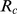. Our model makes the same prediction when considering this specific subset of missense errors. However, when considering the overall missense error rates at AAC and AAU codons due to 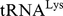, 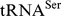, 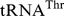, 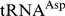, 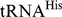, 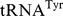 and 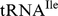 (all one-step neighbors), we come to a very different prediction. Specifically we find that even though AAC has a higher 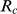 than AAU, it also has a much higher 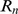 rate. As a result, the *overall* missense error rate for AAC is actually predicted to be higher than AAU. This result illustrates how focusing on only a subset of possible missense errors at a codon, as all previous experiments have done, provides an incomplete and potentially misleading picture.

In contrast to missense error rates, our model predicts 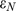 will consistently decline with an increase in 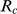, suggesting that nonsense errors may be playing a larger role in driving CUB than commonly accepted [14].

### Intra- and inter-specific variation in the relationship between elongation and error rates

**Figure 4:**
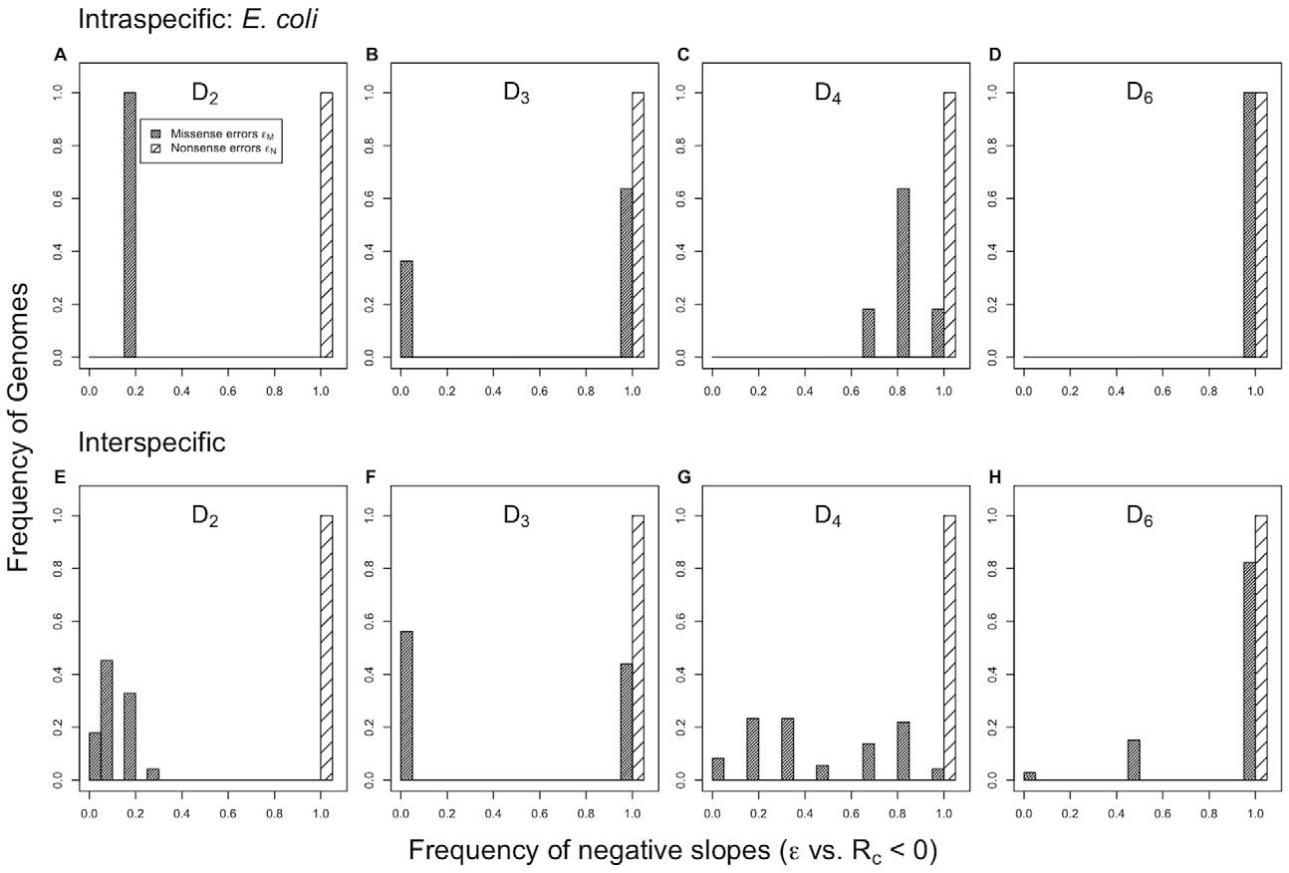
Frequencies of negative relationships between cognate elongation rate 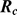 and translation errors 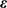. Panels (A–D) represent the distribution of *E. coli* strains that show amino acid specific negative relationship between 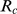 and 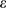, while panels (E–H) represent the distribution of 73 genomes for the same. Amino acids in every degenerate class (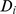) show a negative relationship between cognate elongation rate 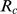 and nonsense error rates (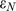) both intra-specifically as well as inter-specifically. A majority of amino acids in the 2-fold degenerate class (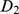) show an increase in missense error rate 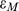 with 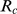 across genomes. As the degeneracy of amino acids increases, we see an increase in the frequency of the expected negative relationship between 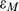 and 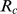 across *E. coli* strains as well as other bacterial species.

In order to evaluate the relationship between cognate elongation rate, 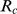, and error rates, we looked across 73 bacterial genomes for inter-specific variation and 11 strains of *E. coli* for intra-specific variation. As before, we categorized amino acids based on the degeneracy of their synonymous codons for each genome. We calculated the fraction of amino acids within each category that showed a *negative* relationship between 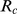 and error rates, 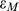 and 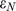 (Figure 4) as expected under the standard model where the abundances of tRNAs are assumed to be uncorrelated.

For both intra- and inter-specific datasets we find that synonymous codons with a higher 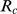 have a lower nonsense error rate 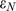 for all amino acids, irrespective of the degenerate class 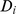 they belong to. However, in the case of missense errors, the relationship between 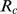 and 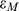 depends on the amino acid degeneracy 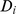 as previously observed in *E. coli* K12 (Figure 3). Amino acids with two synonymous codons (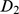) show a strong bias towards a *positive* relationship between 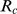 and 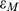, both intra- and inter-specifically (Binomial test, 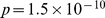 and 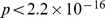, respectively). In the case of isoleucine, the only amino acid in 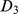, there exists no bias towards a positive or a negative relationship between cognate elongation and missense error rates (Binomial test, intra-specific 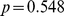 and interspecific 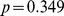). Interestingly 4-fold degenerate amino acids show a bimodal distribution of the fraction of genomes with a negative relationship, and the two 6-fold degenerate amino acids (arginine and leucine) show a strong bias towards negative correlation between 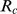 and 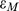 (Binomial test, intra-specific 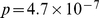 and interspecific 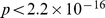). The differences in the relationship between 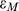 and 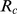 across degenerate classes are similar to the differences in the correlation between 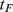 and 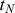 across these classes (Figure 1).

Although the patterns we observe are complex and vary with amino acid degenerate classes, the assumption underlying the standard model that higher cognate tRNA abundance codons will have the lowest translation error rates is predicted to be clearly violated in the case of missense errors – a finding consistent both across bacterial genomes and across various *E. coli* strains. We also find that the positive relationship between missense error rates 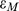 and 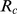 observed within certain amino acids is insensitive to moderate changes in parameter estimates of background nonsense error rates, and wobble parameters (Text S1 B).

## Discussion

For over 30 years, the standard model of translation errors has implicitly assumed that for any given amino acid, the translation error rates are lowest for the codons with the highest tRNA abundances [25], [26], [37]. With respect to missense errors 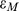, this prediction was based on the implicit and unstated assumption that the distribution of tRNA abundances across the genetic code are uncorrelated. Here we show a consistent positive correlation between the abundance of a tRNA and its one-step mutational neighbors across a wide array of prokaryotes. In order to understand the effects of this relationship on translation errors, we developed a simple model for estimating codon-specific error rates based on the distribution of tRNA gene copy number of a species. Our model takes into account tRNA competition, wobble effects, and intra-ribosomal kinetics of elongation to predict rates of missense and nonsense errors. To our knowledge, ours is the first model to integrate all these factors for estimating translation errors. Using our model, we find that on *average*, both missense and nonsense error rates of a codon decrease with an increase in its cognate tRNA elongation rate. This average behavior is consistent with expectations under the standard model of how codon specific error rates scale with cognate tRNA abundance [12], [15], [25], [38]. However, the expected relationship between error rates and cognate tRNA abundances does not hold at finer scales of individual amino acids, the relevant scale for the evolution of CUB.

For about half of the amino acids (10 out of 21) in *E. coli* K12, synonymous codons that have higher cognate elongation rates 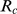 also have higher missense error rates 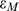. This counterintuitive behavior is due to the fact that tRNA abundances within the genetic code are positively correlated, which leads to an increase in 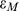 with 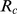, an important pattern that has been overlooked by previous researchers. We find a positive correlation between the abundance of a focal tRNA 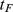 and that of its neighbors 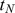 in 69 out of 73 genomes examined here. In addition, the 4 genomes that show a negative 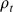 (*E. coli* O157H7, *E. coli* O157H7-EDL933, *Photobacterium profundum* SS9, *Vibrio parahaemolyticus*) also show evidence of a high degree of horizontal gene transfer. Interestingly we also find that the differences in the relationship between 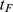 and 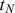 across amino acid degenerate classes is mirrored in the correlation between 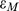 and 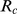. In contrast to 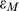, the nonsense error rates 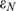 of synonymous codons decrease with an increase in 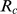 for every amino acid across every genome we analyzed. This is due to the fact that increasing either 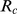 or 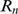 leads to a decrease in ribosomal wait time at that codon which, in turn, leads to a lower 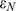. Thus with respect to 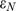, a positive correlation between tRNA abundances actually accentuates the advantage of using codons with higher tRNA abundances. These results lend further support to the hypothesis that nonsense errors play an important but under-appreciated role in the evolution of CUB [11], [39].

The role of tRNA competition has been recognized as an important factor in affecting translation error rates [25], [26], [29]. However, previous studies on the relationship between error rates and tRNA abundances have focused primarily on the effects of modifying cognate tRNA abundances and ignored the effects of near-cognate tRNA abundances. Consistent with our model behavior, [25] showed that when 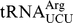 was over-expressed, it led to a decrease in the missense error rate 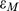 at codons for which the tRNA was a cognate: AGA and AGG. However, if a higher expression level of 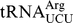 reduces the frequency of 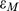 at codons AGA and AGG, why is it not fixed in the population? We argue that increasing the abundance of a given tRNA may not always be adaptive. For instance, over-expressing 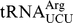 will also lead to an increase in 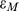 at nearby non-synonymous codons - AAA, ACA, AUA, etc., a testable prediction not considered by [25]. The trade-offs between reducing 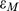 at one codon at the expense of increasing 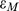 at nearby codons has not been explored. However, these trade-offs likely play an important role in shaping the evolution of tRNA gene copy number and force us to reconsider the evolutionary causes of CUB.

Currently, many researchers believe that selection for translational accuracy, i.e., against missense errors, is a primary force driving the evolution of CUB (see [12], [14], [15], [40]). This belief largely rests on the interpretation of two facts. Firstly, preferred codons are generally those with the highest corresponding tRNA abundances and secondly, sites that are highly conserved and thought to have large effects on protein structure and function, use preferred codons more often than their coding synonyms [12]. Selection for translational accuracy is usually tested using Akashi's test by identifying evolutionarily conserved sites in protein sequences and checking whether they are coded by preferred codons [10], [12], [15], [41]. In light of the above results, we need to revisit the underlying assumptions of Akashi's test [12]. Although, our analysis predicts that a considerable number of amino acids have a positive relationship between missense error rates, 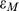 and cognate elongation rates 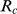, many amino acids in 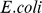 are still predicted to conform to the standard model of lower 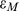 with higher 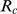. Indeed, in the case of *Drosophila* species used in the original Akashi's paper [12], only 4 out of 21 amino acids are predicted to have a positive relationship between 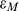 and 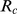. Thus, we argue that the relationship between 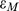 and 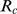 are highly species and amino acid specific and that selection for translation accuracy cannot explain all of the observed CUB at conserved sites. In addition to selection for translational accuracy, selection against nonsense errors [11], [39], [42], mRNA stability [6] and protein misfolding due to ribosome stalling [43], [44] have been shown to affect CUB. In fact, recent evidence suggests that the speed of translating a codon also affects protein folding [43]–[45]. The presence of a codon with a low 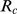, increases the ribosomal waiting time at a codon potentially leading to alternate protein folds. This directly affects the functionality and stability of the protein. Thus, a codon with a higher 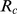 at a conserved site, as observed by Akashi and others, could be under selection to prevent protein misfolding due to an entirely different mechanism unrelated to missense errors. Thus, we would like to stress that the definition of preferred codons used in the Akashis test is based on the genome-wide frequency of codon usage and not on any fundamental biological process. Although, we do not dispute the fact that certain codons are preferred over others at conserved sites, we simply point that the presence of these preferred codons at conserved sites cannot be explained entirely by selection against missense errors and that other selective forces must be responsible for the maintenance of these codons.

CUB often increases with gene expression, such that highly expressed genes tend to use codons with a higher cognate elongation rate 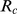 [11], [35], [46]. Thus, these genes would have lower nonsense error rates and wait times, but not necessarily lower missense error rates. This might appear paradoxical, as the failure to minimize missense error rate would presumably increase the probability that a translated protein would be rendered nonfunctional and be selected against. However, the deleterious effects of a high missense error rate can be mitigated by an increased robustness of highly expressed genes. According to [40], [47], [48], highly expressed genes are expected to evolve at a slower rate and also be extremely functionally robust to missense errors. If this is the case, then missense errors in highly expressed genes may not have much of an effect on protein function. These genes maybe perfectly poised for trading off an elevated missense error rate for faster elongation and fewer nonsense error rates.

When it comes to mitigating the effects of non-synonymous mutations and missense errors, the genetic code has been described as “one in a million” [17]. This is due to the fact that amino acids with similar chemical properties are in a genetic ‘neighborhood’, thus reducing the phenotypic effect of any point mutation or missense error. However, unlike point mutations, the frequency of missense errors depends on the distribution of tRNA within the genetic code. The distribution of tRNA abundances is usually attributed to the coevolution between codon usage and tRNA abundances [49]–[51]. However, these studies have not taken into account how changes in tRNA abundances affect the rate of translation errors at neighboring codons. The degree to which the distribution of tRNA abundances within the genetic code is adapted to minimize translation errors remains largely unexplored. Our work suggests that understanding the trade-offs between missense and nonsense errors would provide significant insights into the evolution of tRNA abundances within the genetic code. We believe building mechanistic models of translation errors, as shown here, will help further our understanding of the evolution of tRNA abundances across the genetic code.

## Methods

### tRNA competition

Assuming an exponential waiting process and simple diffusion, the rates at which cognate and near-cognate tRNAs enter the ribosomal *A*-site will be proportional to their abundances. As a result, translation error rates of a codon will depend, in part, on the relative abundances of its cognate and near-cognate tRNAs [25]. Following [8], [31], [32], we use the GCN of a tRNA as a proxy for its abundance.

### Intra-ribosomal dynamics

Discrimination between cognate, near-cognate and non-cognate tRNAs takes place in the peptidyl transfer step of elongation. Since the underlying process is stochastic, there is a non-zero probability that when a cognate tRNA enters the *A*-site it will be rejected or a near-cognate tRNA will be accepted [27]. These probabilities are a function of the kinetic rate constants of various steps involved within the peptidyl transfer and translocation processes during tRNA elongation for both cognate and near-cognate tRNAs [27], [52], [53] (Text S1 A). Based on the rate constants for cognate and near-cognate tRNAs from [27] and equations from [29], we estimated the probability of elongation of a codon by a cognate and near-cognate tRNA per tRNA entry into the ribosomal *A*-site to be 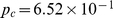 and 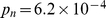, respectively (Text S1 A).

### Wobble effects

One of the factors affecting the rate constants in the intra-ribosome kinetic model described above, is the effect of codon-anticodon wobble. [27] proposed that a wobble mismatch between a codon and its cognate tRNA anticodon, will affect its kinetic rate constants (Text S1 A) and consequently reduce the probability of elongation by that tRNA. Based on [34], [36], we assume that a purine-purine or pyrimidine-pyrimidine wobble reduces the probability of a cognate tRNA being accepted 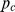, by 40%. This reduction in 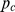 is consistent with estimates based on the kinetic rate constants estimated by [54] for 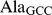 codon that is recognized by 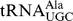 through a pyrimidine-pyrimidine wobble. Similarly, based on [36] ,we assume that a non-canonical purine-pyrimidine wobble (GU/AC) would reduce 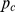 by 36%.

In addition, some codons can be recognized by cognate tRNAs through a non-standard wobble as described by [55], [56]. For instance, C-U and C-A anticodon-codon interactions are considered nonstandard owing to their stereochemistry and thermodynamic constraints. Hence, even though anticodon 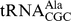 does not lead to a missense error when translating the codon 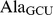, it is considered nonstandard translation due to its C-U wobble. We call these tRNAs ‘pseudo-cognates’. We assume that the probability of elongation of a codon by pseudo-cognates 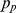 is the same as that of near-cognate tRNAs, i.e., 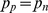.

### Estimation of cognate and near-cognate elongation rates

In order to predict per codon missense and nonsense error rates, we calculated the rates of elongation by cognate and pseudo-cognate tRNAs vs. near-cognate tRNAs at each codon. The cognate elongation rate for codon 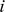 is given by
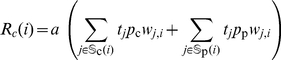
(3)where 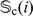 is the set of cognate tRNAs for codon 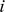, 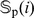 represents the set of pseudo-cognate tRNAs, 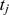 represents the gene copy number of 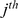 tRNA species, and 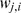 is the reduction in elongation probability due to wobble mismatch.

Similarly, the rate at which near-cognate tRNAs elongate codon 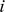 is given by
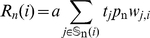
(4)where 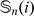 is the set of near-cognate tRNAs with respect to codon 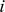. The parameter *a* represents a scaling constant between tRNA gene copy number GCN and elongation rate. For *E. coli*, we used a value of 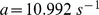, so that the harmonic mean of elongation rates of all codons was 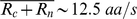 [20], [26], [57].

We assume that nonsense errors occur primarily due to spontaneous drop-off of ribosomes at a given codon when it is waiting for a tRNA. As a result, the nonsense error rate due to spontaneous ribosomal drop-off, 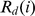, is codon independent and occurs at a constant rate. [24] measured a nonsense error rate of 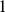 per 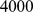 codons. If we assume 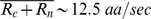, then the background rate of nonsense errors is 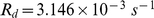.

## Supporting Information

Figure S1 Correlation between a focal tRNA's abundance t_F_ and the abundance of its neighbors t_N_ across prokaryotic genomes. Panel (a) represents the correlation between t_F_ and t_N_ across all amino acids for *B. subtilis*, *E. coli* and *V. parahaemolyticus*. Regression line between t_F_ and t_N_ for *B. subtilis*, *E. coli* and *V. parahaemolyticus* are represented by solid, dashed and dotted lines, respectively. Panel (b) shows the distribution of correlation coefficients ρ_t_ between t_F_ and t_N_ across 73 prokaryotic genomes. About 69 out of 73 genomes (Binomial test, p<10^−15^) have a positive relationship between t_F_ and t_N_.
(1.10 MB EPS)

Figure S2 Contour plot of missense error rates log_10_ (ε_M_) with cognate *R_c_* and near-cognate *R_n_* elongation rates. The black dots represent log_10_(ε_M_) of codons in *E. coli*. Blue dots are the two codons of amino acid asparagine (N). In the case of asparagine, the codon with a higher *R_c_* has a higher ε_M_ as it also has a much higher *R_n_*. The regression line between observed *R_c_* and *R_n_* in *E. coli* is represented as a solid red line. The positive correlation between *R_c_* and *R_n_*, explains why codons with higher *R_c_* sometimes have a higher missense error rate.
(1.69 MB TIF)

Figure S3 The distribution of correlation coefficients between a focal tRNA's abundance t_F_ and the abundance of its neighbors t_N_, ρ_t_. Open bars represents the null distribution of ρ_t_ when tRNAs are randomly distributed across the genetic code, taking into account stereochemical constraints on possible tRNA anticodon types. Red bars represent the observed distribution of ρ_t_ across all 73 prokaryotic genomes. The observed distribution is significantly different from the null distribution (*p*<0.001) across all three degenerate classes.
(1.65 MB EPS)

Figure S4 The distribution of correlation coefficients between a focal tRNA's abundance t_F_ and the abundance of its neighbors t_N_, ρ_t_. Open bars represents the null distribution of ρ_t_ when tRNAs are randomly distributed across the genetic code, taking into account stereochemical constraints on possible tRNA anticodon types as well as the observed amino acid frequency distribution in *E. coli* genome. Red bars represent the observed distribution of ρ_t_ across all 73 prokaryotic genomes. The observed distribution is significantly different from the null distribution (*p*<0.001) across all three degenerate classes.
(1.65 MB EPS)

Figure S5 Correlation of translation error rates ε with cognate elongation rate R_c_ using empirical estimate of tRNA abundances. We find that rates of both (a.) missense ε_M_ and (b.) nonsense errors ε_N_ are negatively correlated with the rate of elongation by cognate tRNAs at that codon. The dashed line indicates the regression line between R_c_ and ε. These results are consistent with the results obtained using tRNA gene copy numbers as proxies for tRNA abundances.
(1.29 MB EPS)

Table S1 List of genomes analyzed.
(0.03 MB PDF)

Table S2 List of codon-specific tRNAs, elongation rates and error rates in *E. coli*.
(0.03 MB PDF)

Text S1 (A) Estimating probability of elongation at a codon during one tRNA insertion attempt. (B) Parameter sensitivity.
(2.44 MB PDF)

## References

[1] Lobley GE, Milne V, Lovie JM, Reeds PJ, Pennie K (1980). Whole body and tissue protein synthesis in cattle.. Br J Nutr.

[2] Pannevis MC, Houlihan DF (1992). The energetic cost of protein synthesis in isolated hepatocytes of rainbow trout (oncorhynchus mykiss).. J Comp Physiol B, Biochem Syst Environ Physiol.

[3] Warner JR (1999). The economics of ribosome biosynthesis in yeast.. Trends Biochem Sci.

[4] Akashi H, Gojobori T (2002). Metabolic efficiency and amino acid composition in the proteomes of *Escherichia coli* and *Bacillus subtilis*.. Proceedings of the National Academy of Sciences of the United States of America.

[5] Sharp PM, Li WH (1986). An evolutionary perspective on synonymous codon usage in unicellular organisms.. J Mol Evol.

[6] Bulmer M (1991). The selection-mutation-drift theory of synonymous codon usage.. Genetics.

[7] Berg OG, Kurland CG (1997). Growth rate-optimised tRNA abundance and codon usage.. Journal of Molecular Biology.

[8] Kanaya S, Yamada Y, Kudo Y, Ikemura T (1999). Studies of codon usage and tRNA genes of 18 unicellular organisms and quantification of *Bacillus subtilis* tRNAs: gene expression level and species-specific diversity of codon usage based on multivariate analysis.. Gene.

[9] Rocha EPC (2004). Codon usage bias from tRNA's point of view: redundancy, specialization, and efficient decoding for translation optimization.. Genome Research.

[10] Drummond DA, Wilke CO (2009). The evolutionary consequences of erroneous protein synthesis.. Nat Rev Genet.

[11] Gilchrist MA, Shah P, Zaretzki R (2009). Measuring and detecting molecular adaptation in codon usage against nonsense errors during protein translation.. Genetics.

[12] Akashi H (1994). Synonymous codon usage in *Drosophila melanogaster*: natural selection and translational accuracy.. Genetics.

[13] Akashi H (2001). Gene expression and molecular evolution.. Current Opinion in Genetics & Development.

[14] Arava Y, Boas FE, Brown PO, Herschlag D (2005). Dissecting eukaryotic translation and its control by ribosome density mapping.. Nucleic Acids Research.

[15] Stoletzki N, Eyre-Walker A (2007). Synonymous codon usage in *Escherichia coli*: selection for translational accuracy.. Molecular Biology and Evolution.

[16] Grantham R (1974). Amino acid difference formula to help explain protein evolution.. Science.

[17] Freeland SJ, Hurst LD (1998). The genetic code is one in a million.. J Mol Evol.

[18] Freeland SJ, Knight RD, Landweber LF, Hurst LD (2000). Early fixation of an optimal genetic code.. Molecular Biology and Evolution.

[19] Higgs P (2009). A four-column theory for the origin of the genetic code: tracing the evolutionary pathways that gave rise to an optimized code.. Biol Direct.

[20] Andersson DI, Bohman K, Isaksson LA, Kurland CG (1982). Translation rates and misreading characteristics of rpsd mutants in *Escherichia coli*.. Mol Gen Genet.

[21] Bouadloun F, Donner D, Kurland CG (1983). Codon-specific missense errors in vivo.. EMBO J.

[22] Precup J, Parker J (1987). Missense misreading of asparagine codons as a function of codon identity and context.. J Biol Chem.

[23] Kurland CG, Ehrenberg M (1987). Growth-optimizing accuracy of gene expression.. Annual review of biophysics and biophysical chemistry.

[24] Jørgensen F, Kurland CG (1990). Processivity errors of gene expression in *Escherichia coli*.. Journal of Molecular Biology.

[25] Kramer EB, Farabaugh PJ (2007). The frequency of translational misreading errors in *e.coli* is largely determined by tRNA competition.. RNA.

[26] Varenne S, Buc J, Lloubes R, Lazdunski C (1984). Translation is a non-uniform process. effect of trna availability on the rate of elongation of nascent polypeptide chains.. Journal of Molecular Biology.

[27] Gromadski KB, Rodnina MV (2004). Kinetic determinants of high-fidelity tRNA discrimination on the ribosome.. Mol Cell.

[28] Ogle JM, Brodersen DE, Clemons WM, Tarry MJ, Carter AP (2001). Recognition of cognate transfer RNA by the 30S ribosomal subunit.. Science.

[29] Fluitt A, Pienaar E, Viljoen H (2007). Ribosome kinetics and aa-tRNA competition determine rate and fidelity of peptide synthesis.. Computational Biology and Chemistry.

[30] Zaher HS, Green R (2009). Fidelity at the molecular level: lessons from protein synthesis.. Cell.

[31] Dong H, Nilsson L, Kurland CG (1996). Co-variation of tRNA abundance and codon usage in *Escherichia coli* at different growth rates.. Journal of Molecular Biology.

[32] Cognat V, Deragon JM, Vinogradova E, Salinas T, Remacle C (2008). On the evolution and expression of *Chlamydomonas reinhardtii* nucleus-encoded transfer RNA genes.. Genetics.

[33] Chan PP, Lowe TM (2009). GtRNAdb: a database of transfer RNA genes detected in genomic sequence.. Nucleic Acids Research.

[34] Lim VI, Curran JF (2001). Analysis of codon:anticodon interactions within the ribosome provides new insights into codon reading and the genetic code structure.. RNA.

[35] Ikemura T (1985). Codon usage and tRNA content in unicellular and multicellular organisms.. Molecular Biology and Evolution.

[36] Curran JF, Yarus M (1989). Rates of aminoacyl-tRNA selection at 29 sense codons in vivo.. Journal of Molecular Biology.

[37] Ikemura T (1981). Correlation between the abundance of *Escherichia coli* transfer RNAs and the occurrence of the respective codons in its protein genes: a proposal for a synonymous codon choice that is optimal for the *e.coli* translational system.. Journal of Molecular Biology.

[38] Drummond DA, Wilke CO (2008). Mistranslation-induced protein misfolding as a dominant constraint on coding-sequence evolution.. Cell.

[39] Gilchrist MA (2007). Combining models of protein translation and population genetics to predict protein production rates from codon usage patterns.. Molecular Biology and Evolution.

[40] Drummond DA, Bloom JD, Adami C, Wilke CO, Arnold FH (2005). Why highly expressed proteins evolve slowly.. Proc Natl Acad Sci USA.

[41] Drummond DA, Raval A, Wilke CO (2006). A single determinant dominates the rate of yeast protein evolution.. Molecular Biology and Evolution.

[42] Gilchrist MA, Wagner A (2006). A model of protein translation including codon bias, nonsense errors, and ribosome recycling.. Journal of Theoretical Biology.

[43] Kimchi-Sarfaty C, Oh JM, Kim IW, Sauna ZE, Calcagno AM (2007). A “silent” polymorphism in the mdr1 gene changes substrate specificity.. Science.

[44] Tsai CJ, Sauna ZE, Kimchi-Sarfaty C, Ambudkar SV, Gottesman MM (2008). Synonymous mutations and ribosome stalling can lead to altered folding pathways and distinct minima.. J Mol Biol.

[45] Marin M (2008). Folding at the rhythm of the rare codon beat.. Biotechnol J.

[46] Greenbaum D, Colangelo C, Williams K, Gerstein M (2003). Comparing protein abundance and mRNA expression levels on a genomic scale.. Genome Biol.

[47] Kellogg E, Juliano N (1997). The structure and function of rubisco and their implications for systematic studies.. American journal of botany.

[48] Wilke CO, Drummond DA (2006). Population genetics of translational robustness.. Genetics.

[49] Wong JT (1975). A co-evolution theory of the genetic code.. Proc Natl Acad Sci USA.

[50] Ardell DH, Sella G (2001). On the evolution of redundancy in genetic codes.. J Mol Evol.

[51] Vetsigian K, Goldenfeld N (2009). Genome rhetoric and the emergence of compositional bias.. Proc Natl Acad Sci USA.

[52] Blanchard SC, Kim HD, Gonzalez RL, Puglisi JD, Chu S (2004). tRNA dynamics on the ribosome during translation.. Proc Natl Acad Sci USA.

[53] Blanchard SC, Gonzalez RL, Kim HD, Chu S, Puglisi JD (2004). tRNA selection and kinetic proofreading in translation.. Nat Struct Mol Biol.

[54] Kothe U, Rodnina MV (2007). Codon reading by tRNA-ala with modified uridine in the wobble position.. Mol Cell.

[55] Agris PF (1991). Wobble position modified nucleosides evolved to select transfer RNA codon recognition: a modified-wobble hypothesis.. Biochimie.

[56] Agris PF, Vendeix FAP, Graham WD (2007). tRNA's wobble decoding of the genome: 40 years of modification.. J Mol Biol.

[57] Sørensen MA, Kurland CG, Pedersen S (1989). Codon usage determines translation rate in *Escherichia coli*.. Journal of Molecular Biology.

