# Supplementary figures and images for: Effect of Correlated tRNA Abundances on Translation Errors and Evolution of Codon Usage Bias
Source: PLoS Genet. 2010 Sep 16;6(9):e1001128. doi: 10.1371/journal.pgen.1001128 (PMC2940732; doi:10.1371/journal.pgen.1001128)

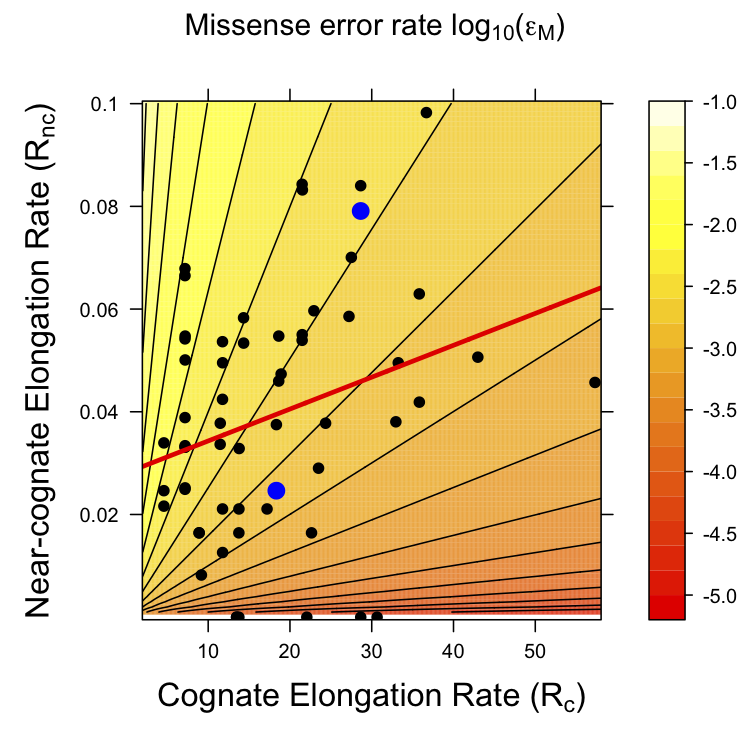

Supplement: Figure S2 — Contour plot of missense error rates log10 (εM) with cognate Rc and near-cognate Rn elongation rates. The black dots represent log10(εM) of codons in E. coli. Blue dots are the two codons of amino acid asparagine (N). In the case of asparagine, the codon with a higher Rc has a higher εM as it also has a much higher Rn. The regression line between observed Rc and Rn in E. coli is represented as a solid red line. The positive correlation between Rc and Rn, explains why codons with higher Rc sometimes have a higher missense error rate. (1.69 MB TIF) [file pgen.1001128.s002.tif]
