# Supplementary material for: Effect of Correlated tRNA Abundances on Translation Errors and Evolution of Codon Usage Bias
Source: PLoS Genet. 2010 Sep 16;6(9):e1001128. doi: 10.1371/journal.pgen.1001128 (PMC2940732; doi:10.1371/journal.pgen.1001128)
Supplement: Table S1 — List of genomes analyzed. (0.03 MB PDF) [file pgen.1001128.s006.pdf]

Table S1: List of Genomes Analyzed

Aeromonas hydrophila ATCC 7966	Aeromonas salmonicida A449
Alkaliphilus metalliredigens QYMF	Alkaliphilus oremlandii OhILAs
Bacillus amyloliquefaciens FZB42	Bacillus anthracis Ames
Bacillus cereus ATCC14579	Bacillus cereus ATCC 10987
Bacillus cereus ZK	Bacillus cereus cytotoxis NVH 391-98
Bacillus subtilis	Bacillus thuringiensis Al Hakam
Bacillus thuringiensis konkukian	Bacillus weihenstephanensis KBAB4
Chromobacterium violaceum	Clostridium beijerinckii NCIMB 8052
Clostridium difficile 630	Clostridium perfringens
Clostridium perfringens ATCC 13124	Colwellia psychrerythraea 34H
Escherichia coli APEC O1	Escherichia coli CFT073
Escherichia coli C ATCC 8739	Escherichia coli E24377A
Escherichia coli HS	Escherichia coli K 12 substr DH10B
Escherichia coli K 12 substr W3110	Escherichia coli O157H7
Escherichia coli O157H7 EDL933	Escherichia coli SMS 3 5
Escherichia coli UTI89	Geobacillus kaustophilus HTA426
Geobacillus thermodenitrificans NG80-2	Heliobacterium modesticaldum Ice1
Klebsiella pneumoniae MGH 78578	Lactobacillus delbrueckii bulgaricus
Lactobacillus delbrueckii bulgaricus ATCC BAA-365	Photobacterium profundum SS9
Pseudoalteromonas haloplanktis TAC125	Psychromonas ingrahamii 37
Salmonella typhimurium LT2	Shewanella ANA-3
Shewanella MR-4	Shewanella MR-7
Shewanella W3-18-1	Shewanella amazonensis SB2B
Shewanella baltica OS155	Shewanella baltica OS185
Shewanella baltica OS195	Shewanella denitrificans OS217
Shewanella frigidimarina NCIMB 400	Shewanella halifaxensis HAW EB4
Shewanella loihica PV-4	Shewanella oneidensis
Shewanella pealeana ATCC 700345	Shewanella putrefaciens CN-32
Shewanella sediminis HAW-EB3	Shewanella woodyi ATCC 51908
Shigella boydii CDC 3083 94	Shigella boydii Sb227
Shigella flexneri 2a	Shigella flexneri 2a 2457T
Shigella flexneri 5 8401	Shigella sonnei Ss046
Symbiobacterium thermophilum IAM14863	Vibrio cholerae
Vibrio cholerae O395	Vibrio fischeri ES114
Vibrio harveyi ATCC BAA-1116	Vibrio parahaemolyticus
Vibrio vulnificus CMCP6	Vibrio vulnificus YJ016
Yersinia pseudotuberculosis IP 31758	
