# Supplementary material for: Effect of Correlated tRNA Abundances on Translation Errors and Evolution of Codon Usage Bias
Source: PLoS Genet. 2010 Sep 16;6(9):e1001128. doi: 10.1371/journal.pgen.1001128 (PMC2940732; doi:10.1371/journal.pgen.1001128)
Supplement: Table S2 — List of codon-specific tRNAs, elongation rates and error rates in E. coli. (0.03 MB PDF) [file pgen.1001128.s007.pdf]

AA	Codon	Cognates	Pseudo-cognates	Near-cognates	R_c	R_n	ε_M	ε_N
A	GCA	UGC	GGC	UCC, UAC, UGA, UGU, UGG, UUC	21.496	5.50E-02	2.55E-03	1.46E-04
A	GCC	GGC, UGC		GAC, GGU, GUC, GCC, GGA, GGG	27.218	5.86E-02	2.15E-03	1.15E-04
A	GCG	UGC	GGC	CGA, CGG, CCC, CGU	13.760	2.11E-02	1.53E-03	2.28E-04
A	GCU	GGC, UGC			22.061	0.00E+00	0.00E+00	1.43E-04
C	UGC	GCA		GCU, GUA, GCC, GGA, GAA, CCA	7.163	5.42E-02	7.51E-03	4.36E-04
C	UGU	GCA		ACG, CCA	4.584	2.16E-02	4.69E-03	6.83E-04
D	GAC	GUC		GGC, GAC, GUA, GUU, GCC, GUG, UUC	21.488	8.43E-02	3.91E-03	1.46E-04
D	GAU	GUC		UUC	13.752	1.64E-02	1.19E-03	2.28E-04
E	GAA	UUC		UGC, UCC, UAC, UUG, GUC, UUU	28.650	8.40E-02	2.92E-03	1.09E-04
E	GAG	UUC		GUC, CCC, CUG	18.336	2.49E-02	1.36E-03	1.71E-04
F	UUC	GAA		GAG, GAC, GCA, GAU, GUA, GGA, UAA, CAA	14.325	5.83E-02	4.05E-03	2.19E-04
F	UUU	GAA		UAA, CAA	9.168	8.21E-03	8.94E-04	3.43E-04
G	GGA	UCC	GCC, CCC	UGC, UAC, UCU, UUC	7.183	5.47E-02	7.56E-03	4.34E-04
G	GGC	GCC, UCC	CCC	GGC, GAC, GCU, GCA, GUC	32.952	3.81E-02	1.15E-03	9.53E-05
G	GGG	CCC, UCC	GCC	CCU, CCG, CCA	11.763	1.26E-02	1.07E-03	2.67E-04
G	GGU	GCC, UCC	CCC	ACG	22.638	1.64E-02	7.25E-04	1.39E-04
H	CAC	GUG		GAG, GUA, GUU, UUG, GUC, GGG, CUG	7.163	6.65E-02	9.20E-03	4.35E-04
H	CAU	GUG		ACG, UUG, CUG	4.584	3.39E-02	7.34E-03	6.81E-04
I	AUA	CAU	GAU	UAG, UAC, UGU, UUU, UCU, UAA, CAU	36.685	9.83E-02	2.67E-03	8.55E-05
I	AUC	GAU	CAU	GAG, GAC, GCU, GUU, GGU, GAA, CAU	21.521	8.32E-02	3.85E-03	1.46E-04
I	AUU	GAU		CAU	13.785	3.28E-02	2.38E-03	2.28E-04
K	AAA	UUU		GUU, UGU, UUG, UCU, UUC	42.976	5.06E-02	1.18E-03	7.31E-05
K	AAG	UUU		GUU, CCU, CAU, CUG, CGU	27.504	7.01E-02	2.54E-03	1.14E-04
L	CUA	UAG	GAG, CAG, UAA	UAC, UUG, UGG	7.189	3.31E-02	4.58E-03	4.35E-04
L	CUC	GAG, UAG	CAG	GAC, GAU, GAA, GUG, GGG	11.477	3.78E-02	3.28E-03	2.73E-04
L	CUG	CAG, UAG	GAG, CAA	CGG, CCG, CAU, CUG	33.243	4.95E-02	1.49E-03	9.45E-05
L	CUU	GAG, UAG	CAG	ACG	8.898	1.64E-02	1.84E-03	3.53E-04
L	UUA	UAA	UAG, CAA	UAC, UGA, GAA	7.171	3.31E-02	4.59E-03	4.36E-04
L	UUG	CAA, UAA	CAG	CGA, GAA, CAU, CCA	11.764	4.95E-02	4.19E-03	2.66E-04
M	AUG	CAU		GAU, CAG, CCU, CAA, CGU	57.301	4.57E-02	7.97E-04	5.49E-05

AA	Codon	Cognates	Pseudo-cognates	Near-cognates	R_c	R_n	ε_M	ε_N
N	AAC	GUU		GCU, GAU, GUA, GGU, GUC, UUU, GUG	28.650	7.91E-02	2.75E-03	1.09E-04
N	AAU	GUU		UUU	18.336	2.46E-02	1.34E-03	1.71E-04
P	CCA	UGG	CGG, GGG	UAG, UGC, UGA, UGU, UUG	7.171	3.34E-02	4.63E-03	4.36E-04
P	CCC	GGG, UGG	CGG	GAG, GGC, GGU, GGA, GUG	11.464	3.37E-02	2.93E-03	2.74E-04
P	CCG	CGG, UGG	GGG	CGA, CAG, CCG, CUG, CGU	11.751	4.24E-02	3.60E-03	2.67E-04
P	CCU	GGG, UGG	CGG	ACG	8.886	1.64E-02	1.84E-03	3.53E-04
Q	CAA	UUG	CUG	UAG, UGG, UUU, GUG, UUC	14.334	5.34E-02	3.71E-03	2.19E-04
Q	CAG	CUG, UUG		CGG, CAG, GUG, CCG	23.493	2.90E-02	1.23E-03	1.34E-04
R	AGA	UCU	CCU	UCC, GCU, UGU, UUU	7.167	3.89E-02	5.39E-03	4.36E-04
R	AGG	CCU, UCU	CCG	GCU, CAU, CC, CCA, CGU	11.751	5.36E-02	4.54E-03	2.66E-04
R	CGA	ACG	UCU, CCG	UAG, UCC, UUG, UGG	17.199	2.11E-02	1.22E-03	1.83E-04
R	CGC	ACG	CCG	GAG, GCU, GCA, GCC, GUG, GGG	18.340	3.75E-02	2.04E-03	1.71E-04
R	CGG	CCG, ACG	CCU	CGG, CAG, CCC, CCA, CUG	24.357	3.78E-02	1.55E-03	1.29E-04
R	CGU	ACG	CCG		28.655	0.00E+00	0.00E+00	1.10E-04
S	UCA	UGA	CGA, GGA	UGC, UGU, UGG, UAA	7.175	2.52E-02	3.50E-03	4.37E-04
S	UCC	GGA, UGA	CGA	GGC, GCA, GUA, GGU, GAA, GGG	18.627	4.60E-02	2.46E-03	1.68E-04
S	UCG	CGA, UGA	GGA	CGG, CCA, CAA, CGU	11.755	2.11E-02	1.79E-03	2.67E-04
S	UCU	GGA, UGA	CGA		13.470	0.00E+00	0.00E+00	2.33E-04
U	ACA	UGU	GGU, CGU	UGC, UGA, UGG, UUU, UCU	7.180	5.01E-02	6.93E-03	4.35E-04
U	ACC	GGU, UGU	CGU	GGC, GCU, GAU, GUU, GGA, GGG	18.631	5.47E-02	2.93E-03	1.68E-04
U	ACG	CGU, UGU	GGU	CGA, CGG, CCU, CAU	18.917	4.74E-02	2.50E-03	1.66E-04
U	ACU	GGU, UGU	CGU		13.474	0.00E+00	0.00E+00	2.33E-04
V	GUA	UAC	GAC	UAG, UGC, UCC, UUC, UAA	35.821	4.19E-02	1.17E-03	8.77E-05
V	GUC	GAC, UAC		GAG, GGC, GAU, GUC, GCC, GAA	35.813	6.29E-02	1.75E-03	8.77E-05
V	GUG	UAC	GAC	CAG, CAU, CCC, CAA	22.929	5.97E-02	2.60E-03	1.37E-04
V	GUU	GAC, UAC			30.656	0.00E+00	0.00E+00	1.03E-04
W	UGG	CCA		GCA, CGA, CCU, CCG, CCC, CAA	7.163	2.49E-02	3.46E-03	4.37E-04
Y	UAC	GUA		GCA, GUU, GUC, GGA, GAA, GUG	21.488	5.39E-02	2.50E-03	1.46E-04
Y	UAU	GUA			13.752	0.00E+00	0.00E+00	2.29E-04
Z	AGC	GCU		GCA, GAU, GUU, GGU, GCC, CCU, UCU	7.163	6.79E-02	9.38E-03	4.35E-04
Z	AGU	GCU		ACG, CCU, UCU	4.584	2.46E-02	5.34E-03	6.82E-04
