# Supplementary material for: Effect of Correlated tRNA Abundances on Translation Errors and Evolution of Codon Usage Bias
Source: PLoS Genet. 2010 Sep 16;6(9):e1001128. doi: 10.1371/journal.pgen.1001128 (PMC2940732; doi:10.1371/journal.pgen.1001128)
Supplement: Text S1 — (A) Estimating probability of elongation at a codon during one tRNA insertion attempt. (B) Parameter sensitivity. (2.44 MB PDF) [file pgen.1001128.s008.pdf]

A Estimating probability of elongation at a codon during one tRNA insertion attempt

The kinetic model of tRNA selection as adapted from Gromadski and Rodnina (2004)

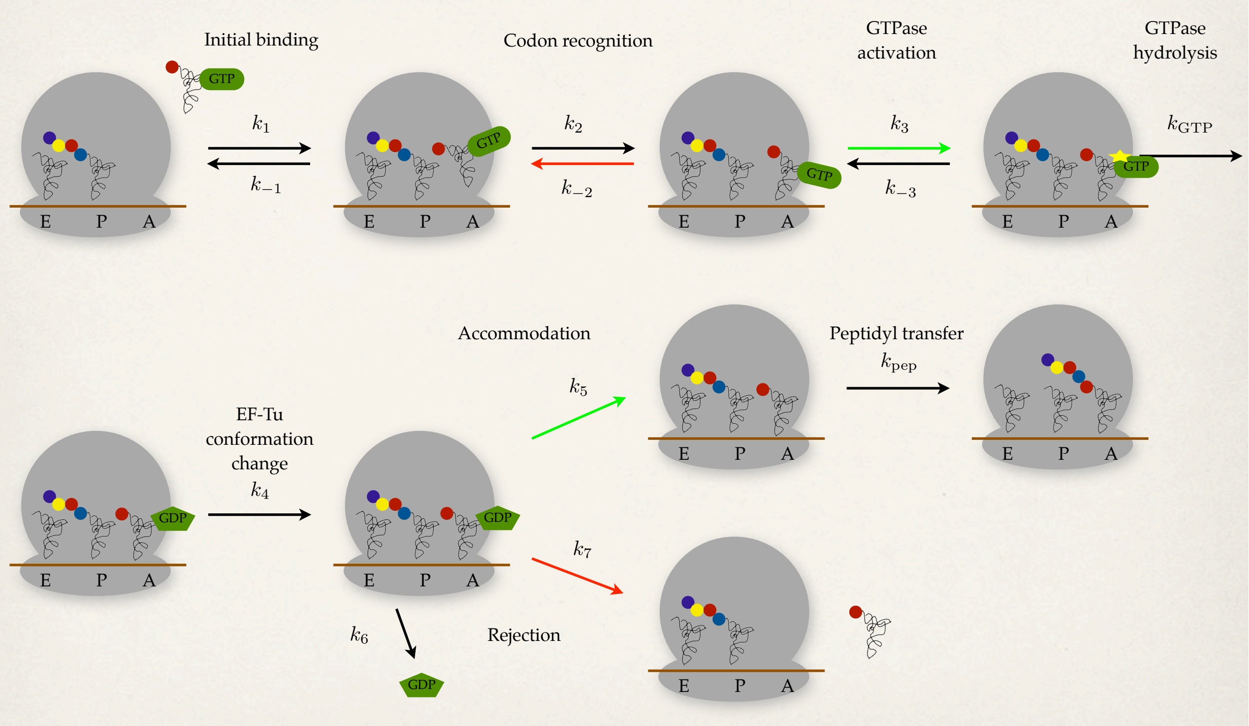

Rate Constant	k_1 (µm^{-1} s^{-1})	k_{-1} (s^{-1})	k_2 (s^{-1})	k_{-2} (s^{-1})	k_3 (s^{-1})	k_{GTP} (s^{-1})	k_4 (s^{-1})	k_5 (s^{-1})	k_7 (s^{-1})	k_{pep} (s^{-1})
Cognate	140	85	190	0.23	260	1000	1000	1000	60	200
Near-cognate	140	85	190	80	0.4	1000	1000	60	1000	200

Using Eqn. (5) from Fluitt, et.al. (2007), we estimated the probability of elongation as

$$p = \frac{P_{23}P_{34}P_{67}}{P_{23}P_{34} + P_{21}} \tag{1}$$

$$P_{23} = \frac{k_2}{k_2 + k_{-1}} \qquad P_{34} = \frac{k_3}{k_3 + k_{-2}} \qquad P_{67} = \frac{k_5}{k_5 + k_7} \qquad P_{21} = \frac{k_{-1}}{k_{-1} + k_2} \tag{2}$$

Plugging in the values for cognate and near-cognate tRNAs, we find $p_c = 6.52 \times 10^{-1}$ and $p_n = 6.2 \times 10^{-4}$.

B Parameter Sensitivity

Since our model was parametrized using empirical data for *E. coli*, we checked for the sensitivity of our analyses to changes in underlying parameters. Specifically, we changed the wobble parameters (w_{RR} and w_{RY}) and the rate of premature termination (R_d). We checked for the sensitivity to parameters by visually comparing the correlation of error rates (ε_M and ε_N) versus cognate elongation rate (R_c) as well as by comparing the distribution of these correlations across amino acids both intra- and inter-specifically.

Cognate elongation rate versus error rates We find no qualitative difference in the relationship between cognate elongation and error rates when the rate of premature termination (R_d) was both increased and decreased by an order of magnitude. However, we did see a corresponding change in the overall nonsense error rate of codons, as expected.

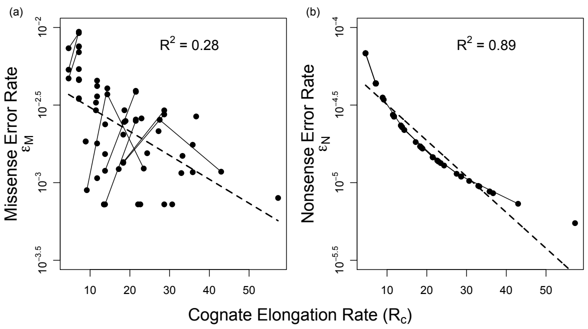

(a) $R_d = 3.146 \times 10^{-4}$

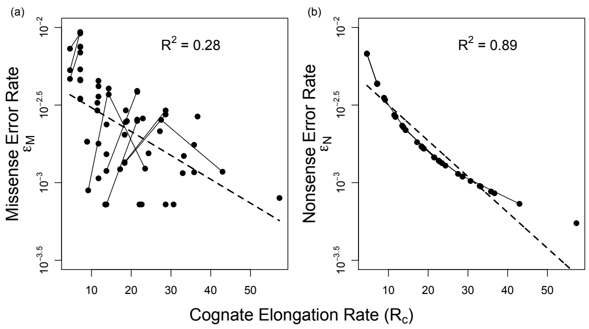

(b) $R_d = 3.146 \times 10^{-2}$

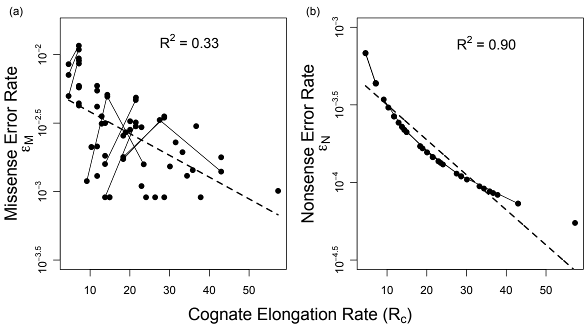

(c) $w_{RR} = 0.8, w_{RY} = 0.64$

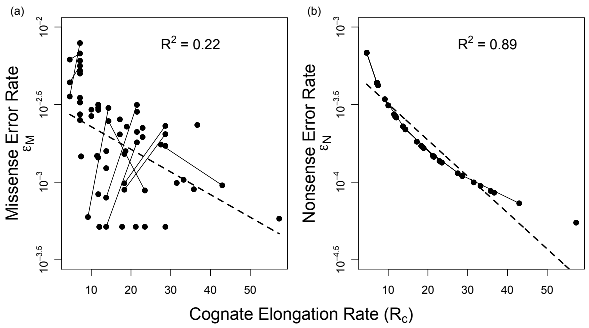

(d) $w_{RR} = 0.4, w_{RY} = 0.64$

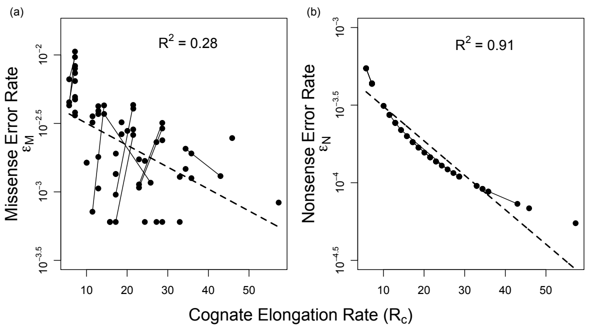

(e) $w_{RR} = 0.6, w_{RY} = 0.8$

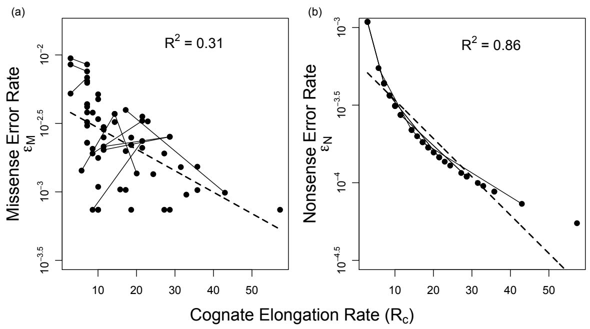

(f) $w_{RR} = 0.6, w_{RY} = 0.4$
